# Assessment and classification of patients with myocardial injury and infarction in clinical practice

**DOI:** 10.1136/heartjnl-2016-309530

**Published:** 2016-11-02

**Authors:** Andrew R Chapman, Philip D Adamson, Nicholas L Mills

**Affiliations:** BHF Centre for Cardiovascular Science, University of Edinburgh, Edinburgh, UK

## Abstract

Myocardial injury is common in patients without acute coronary syndrome, and international guidelines recommend patients with myocardial infarction are classified by aetiology. The universal definition differentiates patients with myocardial infarction due to plaque rupture (type 1) from those due to myocardial oxygen supply-demand imbalance (type 2) secondary to other acute illnesses. Patients with myocardial necrosis, but no symptoms or signs of myocardial ischaemia, are classified as acute or chronic myocardial injury. This classification has not been widely adopted in practice, because the diagnostic criteria for type 2 myocardial infarction encompass a wide range of presentations, and the implications of the diagnosis are uncertain. However, both myocardial injury and type 2 myocardial infarction are common, occurring in more than one-third of all hospitalised patients. These patients have poor short-term and long-term outcomes with two-thirds dead in 5 years. The classification of patients with myocardial infarction continues to evolve, and future guidelines are likely to recognise the importance of identifying coronary artery disease in type 2 myocardial infarction. Clinicians should consider whether coronary artery disease has contributed to myocardial injury, as selected patients are likely to benefit from further investigation and in these patients targeted secondary prevention has the potential to improve outcomes.

## Introduction

The definition of acute myocardial infarction has evolved to accommodate increasingly sensitive markers of myocardial necrosis and imaging methods that allow greater understanding of the pathogenic mechanisms of acute coronary syndrome. As such, the universal definition of myocardial infarction proposes that we classify patients with myocardial infarction based on aetiology.^1^ While this classification has been used in clinical trials to refine primary and secondary endpoints,^2–4^ it has not been widely adopted in clinical practice, and the frequency and implications of subtypes of acute myocardial infarction are uncertain.

We now recognise a spectrum of acute and chronic myocardial injury due to a variety of cardiac and non-cardiac causes in clinical practice. The most contentious diagnosis is that of type 2 myocardial infarction, which is defined as myocardial necrosis with evidence of ischaemia due to myocardial oxygen supply-demand imbalance in the context of another acute illness. Differentiating between patients with type 2 myocardial infarction and those patients with myocardial necrosis in the absence of ischaemia, in whom the recommended classification is myocardial injury, is challenging, as there is considerable overlap between these two clinical entities.^5^ Outcomes for both groups of patients are poor, and investigation and management are inconsistent in practice.^6^ It is likely that this is at least in part due to variability in interpretation of the guidelines.

Here, we summarise the available literature on the prevalence and outcomes of subtypes of myocardial injury and infarction, and aim to provide practical guidance for the clinician to aid the assessment and investigation of patients with undifferentiated myocardial injury.

## Biochemical quantification of myocardial injury

Cardiac troponin is the only recommended biomarker for the detection of myocardial necrosis, and it is integral to the diagnostic criteria for myocardial infarction.^1^ Our ability to accurately measure cardiac troponin has improved through the development of more sensitive assays, with the latest generation high-sensitivity assays capable of detecting cardiac troponin concentrations in the majority of healthy individuals. This has allowed accurate identification of the normal reference range and the 99th centile upper reference limit.^7–9^ The universal definition has recommended the 99th centile as the diagnostic threshold for acute myocardial infarction since 2007, with a rise or fall in cardiac troponin concentrations necessary to confirm the diagnosis^1^ Improvements in assay precision have identified differences in cardiac troponin concentrations between men and women, with the 99th centile twofold lower in women than men across a range of assays.^7^ The use of high-sensitivity cardiac troponin and sex-specific 99th centile upper reference limits increases the diagnosis of myocardial injury and infarction, particularly in women, and identifies a high-risk group of patients with poor outcomes.^8^

There is now widespread adoption of cardiac troponin assays in clinical practice across Europe, with >95% of laboratories using cardiac troponin as the preferred marker for the diagnosis of myocardial infarction.^10^ Over 50% of European laboratories use the 99th centile upper reference limit as the diagnostic threshold; however, as it is 3 years since this survey was undertaken, the proportion today is likely to be higher, given the widespread availability of high-sensitivity cardiac troponin assays and their prominence in national guidelines.

Recent studies have demonstrated that cardiac troponin concentrations below the 99th centile can help in the risk stratification of patients with suspected acute coronary syndrome.^1^ ^8^ ^11–14^ As such, the latest European Society of Cardiology guidelines include additional pathways incorporating lower thresholds of cardiac troponin for risk stratification and earlier testing.^15^ We recently demonstrate in consecutive patients with suspected acute coronary syndrome that a high-sensitivity cardiac troponin I concentration <5 ng/L at presentation had a negative predictive value of 99.6% (95% CI 99.3 to 99.8) for myocardial infarction during the index presentation, or myocardial infarction or cardiac death in 30 days.^16^ Furthermore, patients with troponin concentrations <5 ng/L at presentation had very low rates of adverse cardiac events in 1 year, compared with those with ≥5 ng/L but <99th centile.^16^ These observations now form the basis of our clinical pathway for the assessment of patients with suspected acute coronary syndrome.^17^ The use of cardiac troponin testing in clinical practice is evolving rapidly with cardiac troponin concentrations increasingly used as a continuous measure of cardiovascular risk, rather than simply a binary test to identify those patients with and without myocardial infarction.^16^

## Classification of myocardial injury and infarction

**Figure 1:**
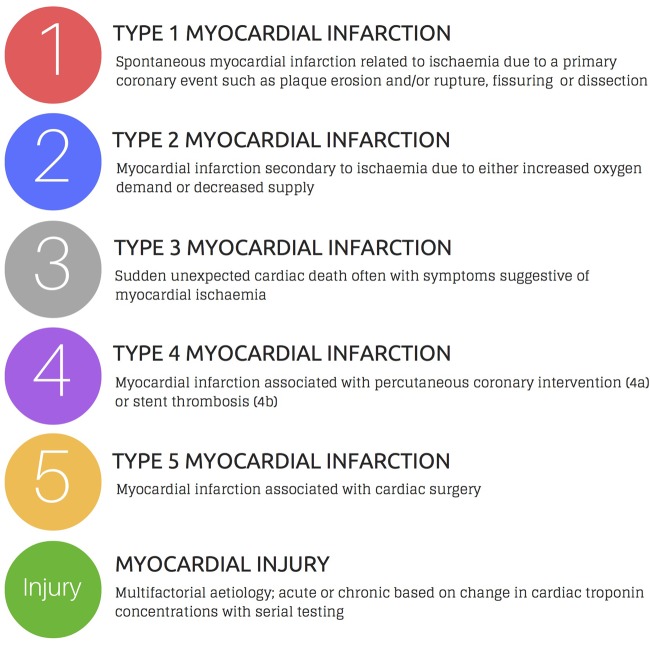
Classification proposed by the third universal definition of myocardial infarction.^1^

The introduction of more sensitive cardiac troponin assays and lower diagnostic thresholds led to a major revision of the guidelines introducing a classification by aetiology to acknowledge that myocardial injury occurs in a wide range of clinical presentations (figure 1). The third universal definition of myocardial infarction provided an international consensus on the classification of myocardial injury and infarction.^1^ The diagnosis of myocardial infarction requires evidence of myocardial necrosis in a clinical setting consistent with acute myocardial ischaemia. These criteria require detection of a rise and/or fall in cardiac biomarker levels (preferably cardiac troponin) with at least one value above the 99th percentile upper reference limit, with at least one of the following: (1) symptoms of myocardial ischaemia, (2) new or presumed new significant ST-segment T-wave changes or new left bundle branch block, (3) development of pathological Q-waves on the electrocardiogram, (4) imaging evidence of loss of viable myocardium or new regional wall motion abnormality or (5) identification of intracoronary thrombus by angiography or autopsy.

The classification distinguishes between type 1 myocardial infarction due to thrombosis of an atherosclerotic plaque and type 2 myocardial infarction due to myocardial oxygen supply-demand imbalance in the context of another acute illness.^1^ Myocardial infarctions presenting as sudden death (type 3), or after percutaneous coronary intervention (type 4) and coronary artery bypass grafting (type 5) are also defined. Acute myocardial injury is classified where troponin concentrations are elevated with evidence of dynamic change in the absence of overt myocardial ischaemia, whereas in chronic myocardial injury troponin concentrations remain unchanged on serial testing. This is an important distinction, as the underlying pathological mechanisms in acute and chronic myocardial injury are likely to differ.

This classification is contentious and was based on expert consensus rather than evidence from prospective clinical trials. While it has been adopted in research studies, implementation in clinical practice has been less consistent. The most contentious diagnosis is that of type 2 myocardial infarction; a concept based on clinical hypothesis and observation without prospective mechanistic evaluation. Patients classified with type 2 myocardial infarction are heterogeneous and have myocardial ischaemia secondary to a variety of acute medical or surgical conditions. Based on the current criteria, a diagnosis of type 2 myocardial infarction could be applied to patients without coronary artery disease. At present, there is no guidance or consensus on the optimal cardiac investigation, management or treatment strategy for patients with type 2 myocardial infarction.

The global task force is reviewing the universal definition of myocardial infarction and recognises the need to provide clearer diagnostic criteria and guidance.^18^ Based on the current guidelines, differentiating between patients with type 2 myocardial infarction and acute myocardial injury is challenging as there remains overlap between these two clinical entities, and classification is therefore inconsistent in clinical practice.^19^ ^20^ Similarly, in the absence of an accepted definition, it is difficult to perform standardised evaluations across different healthcare settings, or to conduct randomised trials to determine the effectiveness of investigative strategies or preventative treatments for these patients.

## Mechanisms of myocardial injury

The majority of cardiac troponin is intracellular, with >90% of troponin isoforms located within the sarcomere, and the remainder unbound within the cytoplasmic pool.^21^ The mechanisms of cardiac troponin release into the circulation are thought to include myocyte necrosis, apoptosis, formation and release of membranous blebs, increased membrane permeability and release of proteolytic troponin degradation products.^21^

It is now recognised that cardiac troponin may be released out with the context of myocardial ischaemia and necrosis, with several purported mechanisms. Cardiomyocytes undergo mechanical stretch in response to pressure or volume overload, and this may trigger activation of intracellular proteases associated with intracellular degradation of troponin.^22^ Furthermore, there is evidence that tachycardia may stimulate stress-responsive integrins within the cardiomyocyte, triggering release of intact cardiac troponin I from viable cardiomyocytes in the absence of necrosis.^23^ Troponin release has also been demonstrated in vivo in patients who develop reversible ischaemia during nuclear perfusion imaging with stress testing. Using an ultrasensitive cardiac troponin I assay with single-molecule counting technology, change in cardiac troponin concentration following stress testing was associated with the extent of myocardial ischaemia.^24^

The universal definition makes a distinction between type 2 myocardial infarction and myocardial injury based on the presence or absence of symptoms and signs of myocardial ischaemia; however, there remains considerable overlap and to date there have been no prospective mechanistic studies to evaluate the range of underlying pathophysiology in these patients. Acute myocardial injury may occur in a variety of cardiac and non-cardiac illnesses (table 1) as a consequence of myocardial oxygen supply-demand mismatch (hypotension, tachycardia or hypoxaemia), or due to direct injury in sepsis or viral myocarditis, or as part of the pathophysiological process in acute left ventricular failure. However, in some cases the presenting illness may be associated with a proinflammatory and prothrombotic state with myocardial injury due to embolisation of platelet aggregates and thrombus from an otherwise silent vulnerable plaque. Furthermore, myocardial injury can occur due to myocardial oxygen supply-demand mismatch in the presence of prognostically important, but unrecognised stable coronary artery disease. It is not, therefore, appropriate to dismiss episodes of acute myocardial injury as a mere bystander phenomenon of no clinical consequence.

**Table 1:** Causes of myocardial necrosis stratified by aetiology

Primary myocardial ischaemia	Supply or demand imbalance causing myocardial ischaemia	Injury not related to myocardial ischaemia	Multifactorial or indeterminate aetiology
Atherosclerotic plaque rupture Intraluminal coronary thrombus Distal microembolisation Coronary artery dissection	Anaemia Aortic dissection Aortic valve disease Tachyarrhythmias or bradyarrhythmias Coronary embolism or vasculitis Coronary endothelial dysfunction Coronary vasospasm Hypertension Left ventricular hypertrophy Hypertrophic cardiomyopathy Respiratory failure Shock Cardiogenic Hypovolaemic Septic	Ablation Cardiac contusion Cardiac surgery Cardiotoxic drugs Cardioversion Cytokine-mediated injury Myocarditis Pacing Rhabdomyolysis	Acute/chronic heart failure Burns Critical illness Infiltrative diseases Amyloidosis Sarcoidosis Pulmonary embolism Pulmonary hypertension Acute kidney injury Chronic kidney disease Strenuous exercise Takotsubo cardiomyopathy Stroke Subarachnoid haemorrhage

Adapted from Thygesen *et al*.^1^

Chronic myocardial injury may occur in structural heart disease (hypertensive heart disease, ischaemic or dilated cardiomyopathy) or secondary to other non-cardiac illnesses such as chronic renal failure. As an example, the detection of chronic myocardial injury may be clinically useful in valvular heart disease, with serum cardiac troponin I concentrations associated with cardiac mass, replacement fibrosis and prognosis in patients with aortic stenosis.^25^ The presence of chronic elevations in cardiac troponin associated with these conditions may contribute to diagnostic uncertainty in patients with suspected acute coronary syndrome. In recognition of this European guidelines for patients with non-ST-segment elevation myocardial infarction only recommends invasive management where a relative change in cardiac troponin concentration of at least 20% can be demonstrated, or where there is at least a fivefold elevation in cardiac troponin concentrations above the 99th centile on presentation.^15^ ^26^

## Incidence of myocardial injury and type 2 myocardial infarction

The introduction of high-sensitivity cardiac troponin assays and lower diagnostic thresholds into clinical practice is likely to result in a disproportionate increase in the number of patients with type 2 myocardial infarction or myocardial injury compared with type 1 myocardial infarction,^8^ ^26^ and may lead to diagnostic uncertainty with the potential for over treatment in patients who do not have acute coronary syndrome.^27–29^

The majority of studies have not used high-sensitivity cardiac troponin assays and therefore may underrecognise the incidence of myocardial injury and type 2 myocardial infarction. The largest reported registry to date was published by Baron *et al*,^30^ who assessed all patients with acute myocardial infarction admitted to hospital in Sweden during 2011 (n=20,138). All diagnoses were classified by the attending clinician, with 88.5% of patients classified as type 1 myocardial infarction and 7.1% as type 2 myocardial infarction. Of note, the frequency of diagnosis of type 2 myocardial infarction varied markedly between centres (0–13%), perhaps illustrating the challenge of consistently applying the current diagnostic classification. In studies which classified all patients with elevated cardiac troponin concentrations, the reported incidence of type 2 myocardial infarction varies between 2% and 37% in unselected hospitalised patients, and from 5% to 71% in patients attending the Emergency Department (table 2).^2^ ^4^ ^31–40^

**Table 2:** Studies reporting incidence of myocardial infarction classified according to the universal definition

				Diagnostic classification (%) proportion of all patients with elevation in baseline cardiac troponin				
	Population	Troponin assay and upper reference limit*	Number with elevated cardiac troponin concentrations (% of total study population)	Myocardial injury (%)	Type 1 MI (%)	Type 2 MI (%)	Type 3/4/5 MI (%)	Unclassified
Javed *et al*^31^	Unselected hospital inpatients with cTnI measured (n=2979)†	cTnI (>40 ng/L) ADVIA immunoassay (Siemens)	701 (23.5%)	461 (65.8%)	143 (20.4%)	64 (9.1%)	9 (1.3%)	24 (3.4%)
El-Haddad *et al*^32^	Unselected hospital inpatients with cTnI measured (n=807)	cTnI (>160 ng/L) Beckman Access	807 (100%)	Not reported	512 (63.4%)	295 (36.6%)	Nil	Nil
Saaby *et al*^33^	Unselected hospital inpatients with cTnI measured (n=4499)	cTnI (>30 ng/L) Architect-STAT (Abbott Diagnostics)	1961 (43.6%)	1408 (71.8%)	397 (20.2%)	144 (7.3%)	12 (0.7%)	Nil
Shah *et al*^6^	Unselected hospital inpatients with cTnI measured (n=2165)	cTnI (>50 ng/L) Architect-STAT (Abbott Diagnostics)	2165 (100%)	522 (24.1%)	1171 (54%)	429 (19.9%)	43 (2%)	Nil
White *et al*^4^	Cardiology inpatients with ACS (2000–2006) (n=2201)	cTnI, cTnT, CK, CK-MB	169 (7.7%)	Not reported	106 (62.7%)	7 (4.1%)	56 (33.2%)	Nil
Szymanski *et al*^34^	Cardiology inpatients with ACS (n=2882)	cTn (not specified)	2882 (100%)	Not reported	2824 (98%)	58 (2%)	Nil	Nil
Stein *et al*^35^	Cardiology and ICU inpatients with ACS (n=2818)	Not reported	2818 (100%)	Not reported	2691 (95.5%)	127 (4.5%)	Nil	Nil
Baron *et al*^30^	Hospital inpatients with ACS (n=19 763)	Not reported	19 763 (100%)	Not reported	17 488 (88.5%)	1403 (7.1%)	141 (0.7%)	731 (3.7%)
Melberg *et al*^36^	Hospital inpatients with ACS (n=1093)	cTnT (>30 ng/L) Roche Elecsys	1093 (100%)	Not reported	967 (88.5%)	17 (1.6%)	109 (9.9%)	Nil
Morrow *et al*^2^	Clinical trial patients with ACS (n=13 608)	Not reported	1218 (8.9%)	Not reported	397 (32.6%)	43 (3.5%)	778 (63.9%)	Nil
Sandoval *et al*^37^	Emergency department patients with cTnI measured (n=1112)	cTnI (>34 ng/L) OCD Vitros	256 (23%)	Not reported	66 (25.8%)	190 (74.2%)	Nil	Nil
Smith *et al*^38^	Emergency department patients with cTnI measured (n=662)	cTnI (>90 ng/L) Siemens Stratus	139 (20.9%)	Not reported	40 (28.8%)	99 (71.2%)	Nil	Nil
Smith *et al*^39^	Emergency department patients with suspected ACS (n=1096)	cTn (not specified)	134 (12.2%)	Not reported	127 (95%)	7 (5%)	Nil	Nil
Bonaca *et al*^40^	Emergency department presentations with suspected ACS (n=381)	cTnI (>100 ng/L) Siemens Centaur	96 (25.2%)	Not reported	86 (90%)	10 (10%)	Nil	Nil
Shah *et al*^8^	Unselected patients with suspected ACS (n=1126)	hs-TnI (F >16 g/L; M >34 ng/L) Architect-STAT high-sensitivity (Abbott Diagnostics)	338 (30%)	40 (11.8%)	242 (71.6%)	56 (16.6%)	Nil	Nil

*All units are standardised to ng/L.

†Twenty-seven exclusions (missing data).

ACS, acute coronary syndrome; CK, creatine kinase; cTnI, cardiac troponin I; cTnT, cardiac troponin T; MI, myocardial infarction.

Differences in the reported incidence may in part be explained by the inconsistent approach to distinguishing type 2 myocardial infarction from acute and chronic myocardial injury across studies. It is perhaps unsurprising that the diagnosis of type 2 myocardial infarction is less frequent in selected populations with acute coronary syndrome.

**Figure 2:**
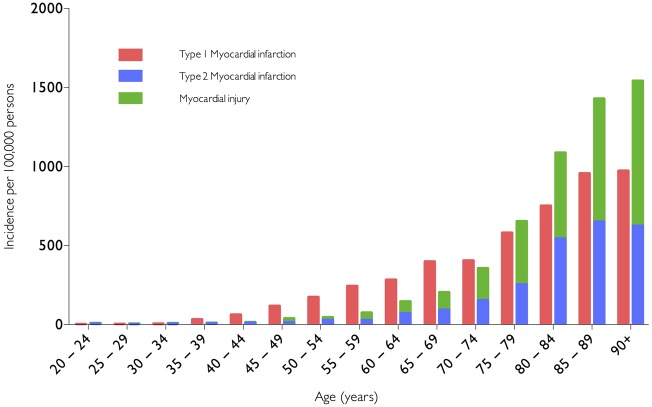
Incidence of myocardial infarction and myocardial injury stratified by age in unselected consecutive hospital inpatients with myocardial necrosis. Reproduced from Shah *et al*.^6^

A previous study at our centre evaluated all patients with elevated plasma cardiac troponin concentrations irrespective of presenting complaint (n=2165), admitted during the validation and implementation of a contemporary sensitive cardiac troponin I assay.^6^ The frequency of type 1 myocardial infarction, type 2 myocardial infarction and myocardial injury was 54%, 20% and 24%, respectively. We demonstrated type 2 myocardial infarction and myocardial injury were as common as type 1 myocardial infarction in clinical practice, and indeed more common than type 1 myocardial infarction in patients ≥75 years of age (figure 2). Lowering the diagnostic threshold with a more sensitive cardiac troponin assay reduced recurrent myocardial infarction or death in patients redefined as having type 1 myocardial infarction, but more than doubled the number of patients with type 2 myocardial infarction or myocardial injury. Despite undergoing additional cardiac investigations, this did not result in changes in treatment, and there was no improvement in clinical outcomes.^6^

Whether adoption of high-sensitivity troponin assays and the 99th centile for diagnosis of myocardial infarction translates into improvements in clinical outcomes for patients with suspected acute coronary syndrome is being evaluated in a stepped wedge cluster randomised trial across Scotland (High-STEACS, NCT: 01852123). If increased sensitivity does not impinge on specificity for the diagnosis of type 1 myocardial infarction, then these assays will improve patient outcomes through better targeting of therapies for coronary artery disease. However, if increased sensitivity leads to poor specificity, then patients may be misdiagnosed and given inappropriate cardiac medications with potentially detrimental outcomes. This trial will establish whether the introduction of high-sensitivity assays into routine clinical practice is detrimental or beneficial to patient management and outcomes; a fundamental and critical assessment for the modern definition of acute myocardial infarction.

## Outcomes of myocardial injury and type 2 myocardial infarction

Patients with type 2 myocardial infarction or myocardial injury have poor clinical outcomes, worse than those patients with type 1 myocardial infarction, with one in three patients dead at 1 year.^6^ In a prospective study of patients with acute coronary syndrome (n=2818), Stein *et al* found an increased risk of death in those with an adjudicated diagnosis of type 2 versus type 1 myocardial infarction at 30 days (13.6% vs 4.9%, p<0.0001) and at 1 year (23.9% vs 8.6%, p<0.0001).^31^ Another single-centre study by El-Haddad reported mortality rates 6.9 times greater in type 2 versus type 1 myocardial infarction at 1 year.^32^

Sarkisian *et al* reviewed 3762 patients who underwent cardiac troponin testing on clinical indication. Patients with acute myocardial injury were at significantly greater risk of all-cause mortality than those with myocardial infarction at a median follow-up of 3.2 years (59% vs 39%, p<0.0001 by log-rank test). In a subgroup analysis, they demonstrate no difference in risk for all-cause mortality between patients with type 2 myocardial infarction or myocardial injury (adjusted hazard ratio (HR) 1.28, 95% CI 0.97 to 1.65).^41^ More recently, we extended follow-up in our cohort of consecutive unselected hospital inpatients,^6^ demonstrating 60% of patients with type 2 myocardial infarction and 75% of patients with myocardial injury were dead at 5 years.^42^ Whether it is possible to improve outcomes in these patients through therapeutic intervention is currently unknown.

The distinction between type 2 myocardial infarction and myocardial injury may, however, be clinically important, as it has been demonstrated that patients classified as having a type 2 myocardial infarction are twice as likely as those with myocardial injury to be readmitted with a type 1 myocardial infarction in 1 year.^6^ This potentially important observation suggests that a proportion of patients with type 2 myocardial infarction may benefit from further investigation and treatment for coronary artery disease. Selection of patients for further investigation requires a greater understanding of the clinical features that identify those patients at increased risk of future acute coronary events and a better understanding of the mechanisms of myocardial injury in this setting.

## Pragmatic classification of patients with myocardial injury and infarction

We believe there remains scope for clarification of the diagnostic criteria for type 2 myocardial infarction and myocardial injury and that this is necessary to encourage clinicians to adopt the classification proposed in the universal definition. This clinical classification acknowledges the central role of coronary artery disease in the pathogenesis of myocardial infarction.

‘Acute myocardial injury’ is a term that clinicians are likely to accept, analogous to ‘acute kidney injury’ or ‘acute liver injury’, and does not predicate the mechanism of injury. This term should embrace all patients with acute myocardial injury identified in the context of an alternative acute illness, including those patients with chest pain or evidence of myocardial ischemia. The mechanism of myocardial injury will determine whether any cardiac or coronary investigations or therapies are indicated.

For example, while a patient with a submassive pulmonary embolism and an elevation in cardiac troponin may have both chest pain and an abnormal electrocardiogram, a diagnosis of type 2 myocardial infarction is unhelpful. The diagnosis is pulmonary embolism and acute myocardial injury due to hypoxia or right ventricular strain; coronary investigations and secondary prevention are not indicated.

The term type 2 myocardial infarction should, in our opinion, be used exclusively in patients with acute myocardial injury where coronary artery disease has contributed and where there may be opportunities to improve outcomes through coronary revascularisation or medical therapy. Selection of patients with acute myocardial injury for further investigation will depend on the nature of primary illness and the patient's probability of having coronary artery disease.

For example, a patient with chronic kidney disease who presents with a community-acquired pneumonia may have persistently elevated cardiac troponin concentrations. The subsequent development of chest pain and ischaemic changes on the electrocardiogram with a temporal rise in serum cardiac troponin concentrations may be due to hypoxia, tachycardia or hypotension, with the acute illness representing a ‘physiological stress test’ identifying otherwise quiescent stable coronary artery disease. The initial diagnosis is ‘acute myocardial injury’, and the need for further investigation for coronary artery disease should be guided by an assessment of cardiovascular risk. In patients with a low probability of coronary artery disease, further cardiac investigations may not be necessary. In patients with an intermediate or high probability, imaging to identify those with coronary artery disease should be considered. Should these investigations confirm the presence of coronary artery disease without evidence of plaque rupture, the diagnosis of type 2 myocardial infarction would be appropriate and secondary prevention should be considered.

Until prospective studies have been performed that define the mechanism of myocardial injury in consecutive patients presenting with an alternative acute illness, clinicians will need to rely on clinical judgement to evaluate the likelihood of coronary artery disease.

## Algorithm for the initial investigation of patients with acute myocardial injury

**Figure 3:**
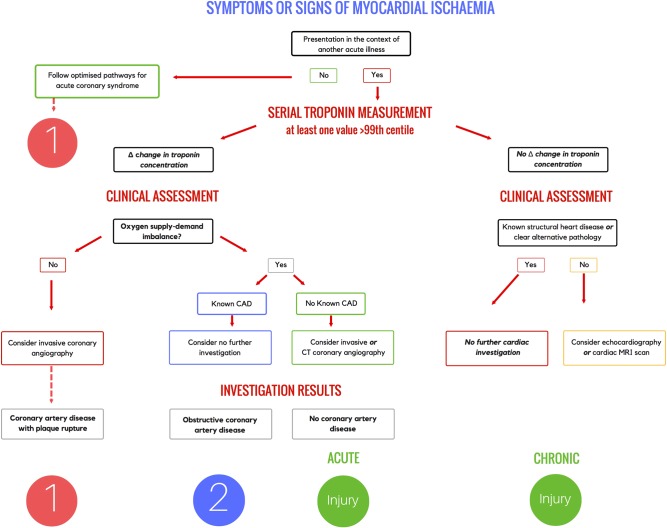
Algorithm for the investigation of patients with elevated cardiac troponin concentrations in the context of an alternative acute illness. Change in cardiac troponin concentration on serial measurement is used to identify patients with acute and chronic myocardial injury. The definition of change in cardiac troponin will be dependent on the assay and should be consistent with the local pathway for the assessment of patients with an isolated presentation with suspected acute coronary syndrome. CAD, coronary artery disease.

We propose a simple decision framework for the initial investigation strategy to determine the aetiology of myocardial injury and identify those with coronary artery disease who may benefit from secondary prevention (figure 3).

**Figure 4:**
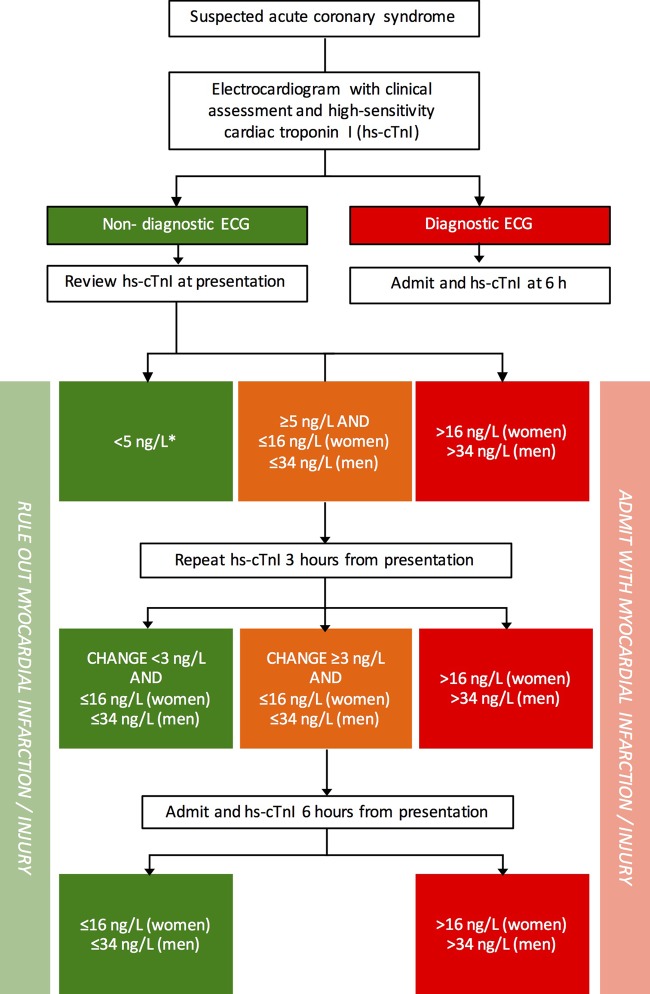
Pathway for the investigation of patients with isolated suspected acute coronary syndrome optimised for the ARCHITECT_STAT_ high-sensitivity cardiac troponin I assay. Reproduced from Shah *et al*.^17^

Patients presenting with isolated symptoms or signs of myocardial ischaemia should be assessed using established pathways for patients with suspected acute coronary syndrome (figure 4). Appropriate diagnostic and risk stratification thresholds will differ depending on the high-sensitivity cardiac troponin assay in use.

Those who develop symptoms and signs of myocardial ischaemia in the context of another acute illness should undergo serial high-sensitivity cardiac troponin testing. Patients should be classified with either acute or chronic myocardial injury based on a change in cardiac troponin concentration, ideally using assay-specific absolute delta criteria. In the absence of these criteria, those with troponin concentrations ≤99th centile at presentation with an increase of >50% of the 99th centile upper reference limit on serial testing (and at least one value >99th centile) are considered to have acute myocardial injury. Where troponin concentrations are >99th centile at presentation, a relative change of >20% is consistent with acute injury.^26^ In patients who meet these criteria, careful clinical assessment is required to determine the likelihood of coronary artery disease. There are no dedicated risk assessment tools for use in this setting, and therefore this assessment relies on clinical judgement, review of the presenting symptoms, medical history, cardiovascular risk factors and serial 12-lead electrocardiographic findings. There is an unmet need for novel risk prediction scores or validation of existing tools, such as the GRACE score, to guide clinicians when assessing patients with acute myocardial injury.

Those patients known to have coronary artery disease may not require further investigation if the mechanism of acute myocardial injury is secondary to oxygen supply-demand mismatch. This may occur in a wide range of conditions where there has been a sustained period of hypotension, tachycardia or hypoxaemia. However, where there is no evidence of oxygen supply-demand mismatch, invasive coronary angiography should be considered to determine whether acute myocardial injury is a consequence of plaque rupture or thrombosis. Where type 1 myocardial infarction is confirmed, application of the GRACE score confers important prognostic information.^15^ Those without known coronary artery disease should be considered for invasive or CT coronary angiography. Where obstructive coronary artery disease is identified and oxygen supply-demand imbalance has been documented, the diagnosis of type 2 myocardial infarction may be helpful and patients should be considered for secondary prevention. Those patients without obstructive coronary artery disease have acute myocardial injury as a consequence of their presenting illness.

Patients with persistently elevated cardiac troponin concentrations without a rise and/or fall on serial sampling are likely to have chronic myocardial injury, which may be due to both cardiac and non-cardiac pathologies. In patients not known to have structural heart disease or a condition known to cause myocardial injury, clinical review should consider whether structural heart disease is likely and guide the need for further cardiac imaging such as echocardiography or cardiac magnetic resonance imaging.

## Conclusions

The implementation of more sensitive troponin assays in clinical practice has increased our awareness of the spectrum of both acute and chronic myocardial injury. While the universal definition classifies myocardial infarction by aetiology, inconsistency in the interpretation and application of these guidelines may be impacting on patient care and outcomes. Identifying patients with acute or chronic myocardial injury, and defining the mechanism of injury is a necessary first step. Careful clinical assessment is necessary to guide the need for further investigations and to identify those patients with coronary artery disease and type 2 myocardial infarction who may benefit from preventative therapies.
